# First report on the enzymatic and immune response of *Metarhizium majus* bag formulated conidia against *Spodoptera frugiperda*: An ecofriendly microbial insecticide

**DOI:** 10.3389/fmicb.2023.1104079

**Published:** 2023-03-02

**Authors:** Vivekanandhan Perumal, Swathy Kannan, Lucy Alford, Sarayut Pittarate, Ruchika Geedi, Dilipan Elangovan, Ramachandran Marimuthu, Patcharin Krutmuang

**Affiliations:** ^1^Department of Entomology and Plant Pathology, Faculty of Agriculture, Chiang Mai University, Chiang Mai, Thailand; ^2^Department of Physiology, Saveetha Dental College and Hospitals, Saveetha Institute of Medical and Technical Sciences, Saveetha University, Chennai, Tamil Nadu, India; ^3^School of Biological Sciences, University of Bristol, Bristol, United Kingdom; ^4^Geedi-Horticultural Insects Research Laboratory, USDA- Agricultural Research Services, Wooster, OH, United States; ^5^Department of Botany, School of Life Sciences, Periyar University, Salem, Tamil Nadu, India; ^6^Innovative Agriculture Research Center, Faculty of Agriculture, Chiang Mai University, Chiang Mai, Thailand

**Keywords:** *Metarhizium majus*, *Spodoptera frugiperda*, *Eudrilus eugeniae*, antioxidant enzyme, microbial insecticide, natural insecticide

## Abstract

Entomopathogenic fungi from microbial sources are a powerful tool for combating insecticide resistance in insect pests. The purpose of the current study was to isolate, identify, and evaluate bag-formulated entomopathogenic fungal conidial virulence against insect pests. We further investigated the enzymatic responses induced by the entomopathogenic fungi as well as the effect on a non-target species. Entomopathogenic fungi were isolated from the Palamalai Hills, India, using the insect bait method, and the *Metarhizium majus* (MK418990.1) entomopathogen was identified using biotechnological techniques (genomic DNA isolation and 18S rDNA amplification). Bag-formulated fungal conidial efficacy (2.5 × 10^3^, 2.5 × 10^4^, 2.5 × 10^5^, 2.5 × 10^6^, and 2.5 × 10^7^ conidia/ml) was evaluated against third instar larvae of *Spodoptera frugiperda* at 3, 6, 9, and 12 days of treatment, and acid and alkaline phosphatases, catalase, and superoxide dismutase enzymatic responses were evaluated at 3 days post-treatment. After 12 days of treatment, non-target assays on the earthworm *Eudrilus eugeniae* were performed using an artificial soil assay. Results of the bag formulated fungal conidial treatment showed that *S. frugiperda* had high susceptibility rates at higher concentrations (2.5 × 10^7^ conidia/ml) of *M. majus*. Lower concentration of 2.5 × 10^3^ conidia/ml caused 68.6% mortality, while 2.5 × 10^7^ conidia/ml caused 100% mortality at 9 days post treatment. Investigation into enzymatic responses revealed that at 3 days post *M. majus* conidia exposure (2.5 × 10^3^ conidia/ml), insect enzyme levels had significantly changed, with acid and alkaline phosphatases, and catalase enzymes significantly reduced and superoxide dismutase enzymes significantly raised relative to the control. After 12 days of treatment, no sublethal effects of *M. majus* conidia were observed on *E. eugeniae*, with no observed damage to gut tissues including lumen and epithelial cells, the nucleus, setae, coelom, mitochondria, and muscles. This study offers support for the use of fungal conidia in the target-specific control of insect pests.

## Introduction

The Fall Armyworm, *Spodoptera frugiperda*, is an insect pest of global economic importance, capable of feeding on over 80 different types of crops, including rice, sorghum, millet, sugarcane, cotton, and vegetable crops (Shylesha et al., 2018; Rwomushana, 2019; Pittarate et al., 2021). Of particular importance to the African continent is maize, on which over 220 million people are reliant on this staple crop. For this reason, *S. frugiperda* represents a major threat to food security. Due to a lack of precise control measures, *S. frugiperda* is responsible for losses ranging from 8.3 to 20.6 million metric tons per year (Xiao et al., 2020; Pittarate et al., 2021). Unfortunately, synthetic chemicals commonly used in the control of insect pests, although initially promising, result in insecticide resistence in the target pest following repeated use (Vivekanandhan et al., 2021). Furthermore, synthetic chemical pesticides pollute the environment and have negative impacts on non-target organisms. As a result, scientists and researchers are looking to new tools in the control of insect pests that are effective, target-specific, efficacious at low concentrations, and are environmentally friendly (Batta, 2003; Vivekanandhan et al., 2022a,b,c).

Entomopathogenic fungi represent one such promosing tool in the control of insect pests and are used against a variety of agricultural insect pests globally (Singh et al., 2017; Kumar et al., 2019; Liu et al., 2021). The pathogenicity of entomopathogenic fungi begins when spores adhere to the insect’s outer cuticle. When conditions are favourable, the conidial spores germinate and penetrate the insect’s cuticle. The fungi subsequently secrete toxic chemical constituents into the insect’s hemolymph when conidia enter the insect host, defeating the insect’s immune system and killing the insect pest (Vivekanandhan et al., 2022c). Of the known entomopathogenic fungi, *Beauveria bassiana*, *Metarhizium anisopliae*, *B. brongniartii*, and *I. fumosorosea* are currently the most commonly used in insect pest control. However, other species including *Nomuraea rileyi*, *Fusarium oxysporum*, and *Verticillium lecanii*, have also been reported to display high virulence in insect pests (Rao et al., 2006; Vivekanandhan et al., 2022a,b), with many species capable of inducing pathogenicity across the different developmental stages (Feng et al., 1994; Wu et al., 2020). The entomopathogenic fungi *B. brongniartii*, for example, has been observed to be affective against larval, pupal and adults stages of *S. litura* under laboratory conditions (Wu et al., 2020), highlighting the potential for entomopathogenic fungal control to target pest insects at various stages of development.

*Metarhizium* spp., an entomopathogenic fungus with a restricted host range, safety, environmental friendliness, and ease of mass production, has been the subject of extensive research (Schrank and Vainstein, 2010; Canassa et al., 2020; Vivekanandhan et al., 2020, 2022a,b; Wasuwan et al., 2021). From the arctic to the tropics, the *Metarhizum* genus is found all over the world. It is a member of the class Hyphomycetes, which reproduces by conidia, or spores (Aw and Hue, 2017). The four original varieties of the *Metarhizium* genus were *M. anisopliae*, *M. taii*, *M. pingshaense*, and *M. guizhouense*. However, the *Metarhizium* genus was reclassified into nine species in 2009 by Bischoff, Rehner, and Humber. These species are *M. anisopliae*, *M. acridum*, *M. guizhouense*, M*. pingshaense*, *M. lepidiotae*, *M. majus*, *M. robertsii*, *M. brunneum*, and *M. globosum* (Thanakitpipattana et al., 2020).

Over the last three decades, a large number of mycoinsecticides based on *Metarhizium*, as well as *Beauveria* species, have been commercialised and registered (Wraight et al., 2001; de Faria and Wraight, 2007; Zimmermann, 2007). In many countries, the spores of *M. anisopliae*, *B. bassiana* and *B. brongniartii* have been successfully formulated as mycopesticides, and several of these products have passed registration requirements and are thus currently widely used in the biocontrol of pest insects (Wraight et al., 2001; de Faria and Wraight, 2007). *L. lecanii* was the first fungus to be developed as an inundated mycoinsecticide for use in medium to large-scale glasshouse farming (Shah and Pell, 2003). Here, the active ingredients from two different isolates were combined to create two commercial products: “Vertalec” for aphid control and “Mycotal” for whitefly and thrip control (Shah and Pell, 2003). Both products are registered in a number of European and non-European countries, including the Netherlands, Finland, Denmark, France, Norway, Turkey, Spain, and the United Kingdom.

When mass-producing entomopathogenic fungi for commercial use, two main methods exist: *via* a liquid culture or *via* a solid culture. Conidial production is faster in liquid cultures (fermentation) than in solid cultures. In addition, blastospores are produced in deep agitated liquid cultures. Although blastospores are more delicate and shorter-lived than conidia, they have the capacity to be as virulent as conidia (Hall, 1981; Bartlett and Jaronski, 1988; Pham et al., 2009). A sophisticated but commercially viable process can be used to create an effective, long-lasting, wettable powder or suspension concentrate of blastospores (Kim et al., 2013). The majority of the fungal-based pesticides, however, are made with conidia from solid cultures. Conidia must be separated from the cereal substrate on which they were cultured after solid culture in order to formulate powder and liquid products. This is a labour-intensive process that ultimately raises production costs. A conidia-harvesting machine (Mycoharvester)^1^, was produced to overcome this issue, however, the cost of production must be reduced if fungal-based pesticides are to compete with chemicals.

The study entomopathogenic fungus species for the current study is *Metarhizium majus*; a species capable of growing in low and medium climatic conditions. Indeed, microbial insecticide formulations based on entomopathogenic fungi *Metarhizium* species are more active at environmental conditions of 20–40°C, with a fungal insecticide life time of 30.8 months (Batta, 2003). *Metarhizium majus* was originally isolated from the edible insect *Protaetia brevitarsis* in Korea (Kwak et al., 2021), although has yet to be thoroughly studied for its use as an insecticidal agent. The current study therefore aimed to isolate, characterize, and bag formulations of the entomopathogenic fungi *M. majus* and investigate the enzymatic and immune responses against *S. frugiperda*. Toxicity effects were further evaluated on the non-target species *Eudrilus eugeniae*. Ultimately, the current study aims to increase current understanding on the potential of *M. majus for use as an* entomopathogenic agent in the target-specific control of insect pests.

## Materials and methods

### Soil sample

Soil samples were collected from the Palamalai Hills, located approximately 25 km north-east from the city centre of Coimbatore, Tamil Nadu, India (11.7355° North, 77.7494° East). Soil samples of 1 kg were collected from a depth of 1–20 cm. Before soil collection, leaf litter and other surface particles were removed according to the method detailed in Vivekanandhan et al. (2020). Collected soil was sealed in a sterile bag and kept at 4°C.

### Entomopathogenic fungi isolation

Insect-borne pathogenic fungi were isolated from the soil using the insect bait method (Vivekanandhan et al., 2021). The insect bait method is a very sensitive technique for entomopathogenic fungi isolation. A total of 10 greater wax moth, *Galleria mellonella*, at the early third instar larvae stage were transferred to a plastic container. The plastic container [11 (L) × 7 (W) × 9 (H) cm], containing 200 g of forest sample soil, was covered with a lid and transferred to the incubator at ambient temperature and relative humidity (27 ± 2°C and 80% RH).

Plastic containers were observed twice a day for 10 days. Every day, dead larvae were collected and sterilised with 70% ethanol for 10 min, and then washed with sterile water. Sterilized insect cadavers were placed on Petri plates (90 × 15 mm) containing PDA medium. Plates were kept at 27 ± 1°C and 85% relative humidity. After 5 days, the pure fungal cultures were separated and stored in a BOD incubator so they could be used in further experiments.

### Morphological confirmation

Isolated entomopathogenic fungi were morphologically identified according to Vivekanandhan et al. (2021), based on the fungal colony, color, mycelium shape, and spores. The morphological characteristics of entomopathogenic fungi were evaluated after lactophenol cotton blue (LCB) staining. The glass slides were observed using a light microscope at 40× magnification (Olympus-CH20i/India).

### Mass culturing of fungi

To isolate genomic DNA from entomopathogenic fungi, liquid cultures were prepared according to the methods detailed in Vivekanandhan et al. (2021). The culturing flask was filled with 150 ml of potato dextrose broth (PDB) and then sterilised at 120°C using an autoclave for 15 min. The liquid medium was transferred to an aseptic condition (LAF) and 1 × 10^6^ conidia/ml was subsequently added to the culture medium. In addition to conidial inoculation, 0.5 ml of chloramphenicol was also added as an anti-bacterial agent. The liquid medium was then mixed and incubated for 7–10 days at 28 ± 1°C to allow for fungal growth.

### Molecular identification

#### DNA extraction

After 7–10 days of incubation, Whatman No. 1 filter paper was used to filter the young fungal mycelium (HiMedia, India) and the fungal mat used to extract genomic genetic material. 0.5 g of fungi biomass was broken down for extraction using liquid nitrogen in a sterile mortar and pestle. After the mycelium had been broken down, 2.5 ml of freshly prepared CTAB lysis buffer was added, and the solution transferred to sterile microtubes. Microtubes were incubated in a water bath at 60°C for 60 min. Following incubation, the microtubes were placed at 4°C for 18 min before being centrifuged for 15 min at 8,500 rpm. After centrifugation, the supernatant was transferred to new tubes with an equal amount of chloroform and isoamyl alcohol at a ratio of 24:1 and lightly shaken until an emulsion appeared. Microtubes were then centrifuged at 13,000 rpm for 20 min and the resultant supernatant poured into new tubes. After mixing an equal amount of ice-cold isopropanol with 90% ethanol, micro-tubes were incubated at 25°C for 1 h. Following incubation, micro-tubes were centrifuged at 13,500 rpm for 20 min to collect the genomic DNA pellet in new tubes. Finally, 70% ethanol was used to clean the genomic DNA. Following removal of the ethanol, the purity of the genomic DNA was checked using 0.8% agarose gel electrophoresis.

#### PCR amplification

For fungal genetic material amplification, following forward (GTAGTCATATGCTTGTCTC) and reverse (CTTCCGTCAATTCCTTTAAG) primers, three virulent entomopathogenic fungal genomic DNA were amplified using the 18 s rDNA primers (NS1 and NS2). PCR amplification was carried out in a 20 μl reaction volume containing 1X PCR buffer (1.5 mM MgCl_2_), 0.2 mM of each dNTP (dATP, dGTP, dCTP, and dTTP), 1 μl genomic DNA, 0.2 μl II DNA polymerase enzyme, 0.1 mg/ml BSA, 3% DMSO, 0.5 M Betaine, and 5 μl primers. For the PCR, the following steps were used: annealing at 50°C for 30 s, elongation at 72°C for 2 min, and extension at 72°C for 7 min.

#### Fungal sequence examination

At Chromous Biotech Pvt. Ltd. in Tamil Nadu, India, the DNA was sequenced, and the sequence was then submitted to GenBank. By using BLAST analysis, sequence results were compared to the GenBank data bases. Results from the NCBI Genbank database were used to identify the entire species. Following that, the CLUSTAL W programme was used to align the order of the organisms with IN-5. Use of CLUSTALW (BioEdit) (Hall, 1999) for multiple sequence alignments. MegAlign was used to determine the nucleotide and deduced amino acid sequence homology (DNA Star, Inc., Madison, WI, United States). For phylogenetic analysis, FigTree V1.3.1 software was used, along with MEGA5 (Tamura et al., 2011), distance matrix, neighbor-joining (Saitou and Nei, 1987), and maximum parsimony methods (Tamura et al., 2004; Rambaut, 2009).

#### Rearing of insect culture

*Spodoptera frugiperda* 1st instar larvae were obtained from a colony established in the laboratory and the date it was established and from where the insects were collected was provided (Pittarate et al., 2021). Larvae were individually cultured on maize in a small plastic cup and maintained under laboratory conditions. All experiments were conducted in the laboratory at 28 ± 1°C and 75–85% relative humidity with a photoperiod of 12L: 12 D.

#### Non-target species rearing

Earthworms, *E. eugeniae,* were reared using a cow dung and agricultural waste (3:1) ratio in plastic containers [30 (L) × 20 (W) × 20 (H) cm] at a temperature of 28 ± 1°C.

#### Solid culture of fungi on maize

*Metarhizium majus* fungal conidia were grown in a polyethylene bag using maize (500 g). To reduce bacterial contamination, maize (300 g) was placed in a polyethylene bag, and then 100 ml distilled water containing 1 ml citric acid (50% stock solution) was added to the bag. The bag was placed in a water bath at 90 ± 1°C for 1 h before being autoclaved at 12L°C for 15 min. After cooling to room temperature, each bag was inoculated with a 5-ml aliquot of fungal isolate liquid culture. For 2 weeks, the inoculated bags were kept at 28 ± 1°C and a photoperiod of 16L: 8 D. The mycotized maize was dried at room temperature for 2 days to reduce the moisture content to 5%, as measured by a moisture analyser.

#### Screen bag formulation

**Figure 1:**
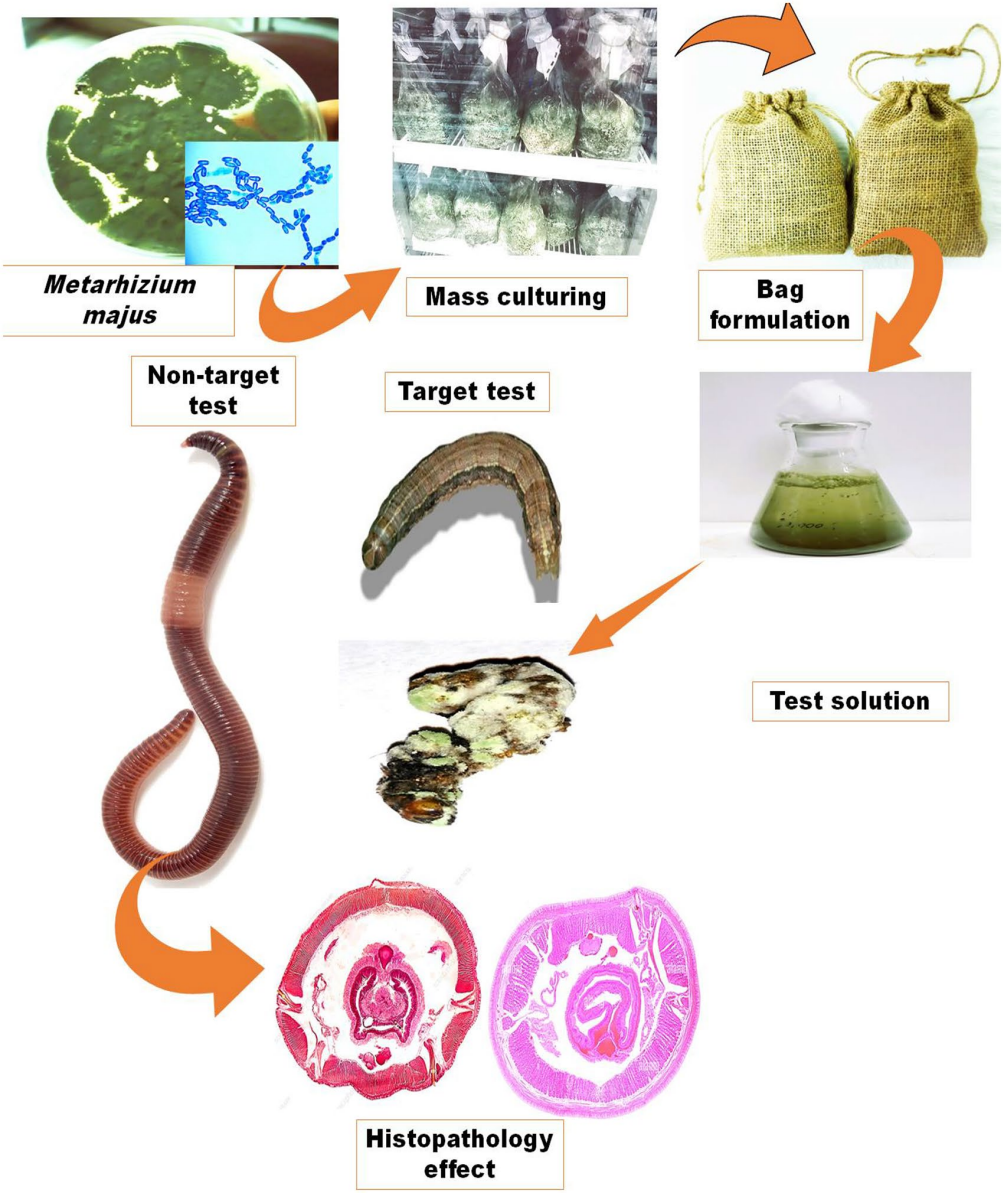
Production of an entomopathogenic fungi *M. majus* conidia bag formulation. The formulation preparation process and how it is used to make a fungal suspension for spray application are depicted schematically.

The fungal granule containers were made of a burlap bag (10 × 14 cm^2^) with a pore diameter of 15 m (Alibaba.com, Bangkok, Thailand). To make a screen bag formulation, dried fungal granules (50 g) were packed in the screen bag and closed at the top. To store the finished product, the bag was wrapped in aluminium foil. To make a conidial suspension, the screen bag was immersed in 5 l of distilled water and then agitated to extract the conidia into a 50 ml fresh centrifuge tube (Figure 1). The conidia were then vortexed vigorously for 1 min to homogenise before sieving. The conidial homogenate suspensions were sieved through four layers of sterile cheesecloth and placed in sterile centrifuge tubes to be used later. The concentrations of conidia were measured with a Neubauer hemocytometer under a bright-field microscope (Olympus SZ51/SZ61, India). For the bioassay, five concentrations of 2.5 × 10^3^, 2.5 × 10^4^, 2.5 × 10^5^, 2.5 × 10^6^, and 2.5 × 10^7^ conidia/ml were prepared in dilutions.

#### Bioassay

The concentrations of the fungal solution (2.5 × 10^3^, 2.5 × 10^4^, 2.5 × 10^5^, 2.5 × 10^6^, and 2.5 × 10^7^ conidia/ml) were prepared as mentioned above. All the entomopathogenic fungi spore concentrations were prepared in deionized water containing 0.02% Tween 80.

#### Larvicidal bioassay

A fungal virulence test was done on the third instar larvae of *S. frugiperda* by using the maize dip technique. Five different concentrations were prepared from the test solution and were as follows: 1 × 10^5^, 1 × 10^6^, 1 × 10^7^, 1 × 10^8^ and 1 × 10^9^ conidia/ml. The maize was individually dipped in various concentrations of *M. majus* fungal conidia. In the test solution, the maize was dipped for 5 s then air dried and placed into a Petri dish. Each Petri dish contained 15 third instar larvae of *S. frugiperda*. Distilled water was used as a negative control. The larval mortality rate was recorded following 3, 6, 9, and 12 days of treatment. All experiments were conducted in triplicate. The percentage mortality was calculated every 3 days post treatment using Abbott’s formula (Abbott, 1925) as shown below:

$\mathrm{Percentage}\;\;\mathrm{mortality}=\frac{\mathrm{Number}\;\mathrm{of}\;\mathrm{dead}\;\mathrm{larve}\;\mathrm{in}\;\mathrm{treatment}}{\mathrm{Total}\;\mathrm{number}\;\mathrm{of}\;\mathrm{larvae}\;\mathrm{introduced}}\times 100$

$\begin{array}{l} \mathrm{Corrected}\;\mathrm{mortality}\\ =\frac{\mathrm{Observed mortality in treatment}-\mathrm{Observed}\;\mathrm{mortality}\;\mathrm{in}\;\mathrm{control}\;}{100-\mathrm{Control}\;\mathrm{mortality}}\\ \times 100 \end{array}$

#### Insect enzyme homogenate preparation

Larval midgut tissue was homogenized with 2 ml of PBS buffer and spun at 4°C, 10,000 rpm for 15 min. Cell debris and solid waste were removed, and the supernatant was poured into a clean centrifuge tube, put on ice, and used straight away for enzyme assays.

#### Acid and alkaline phosphatase assays

Acid and alkaline phosphatase (ACP and ALP) levels were determined using the procedure described by Asakura (1978), with minor modifications. Acid phosphatase activity was determined by thoroughly mixing 0.05 ml of an enzyme source with 2 ml of alkaline buffer solution. The tubes were then incubated for 30 min at 36°C. 2 ml of NaOH solution was added and thoroughly mixed. The absorbance was then measured in a spectrophotometer at 405 nm.

In the alkaline phosphatase activity, 50 mM of sodium acetate buffer (pH; 4.6) was used in place of the alkaline buffer. For 20 μl larvae midgut tissue homogenates, a 500 μl sample of 50 mM Tris–HCl buffer (pH 8.0) was prepared and mixed with an equal volume of the respective buffer containing 12.5 mM p-nitrophenyl phosphate. The enzymatic reaction was stopped after 15 min at 37°C in a water bath by adding 0.5 N NaOH solutions, and subsequently centrifuged (4,000 ×*g*; 5 min). The absorbance of the resulting clear supernatants was measured at 405 nm.

#### Catalase assay

Catalase (CAT) activity was measured using the method by Wang et al. (2001). 2.9 ml of Solution-A (50 mM KPO_4_; pH 7.0) and Solution-B (0.036% H_2_O_2_, KPO_4_), and 0.03 ml of H_2_O_2_ mixed with phosphate buffer, were mixed with 0.1 ml of larvae midgut tissue homogenate and read against control cuvette containing H_2_O_2_ and phosphate buffer only. Absorbance was recorded at 240 nm.

#### Superoxide dismutase assay

The method of Marklund and Marklund (1974) was used to test superoxide dismutase assay (SOD) activity. 50 μl of a homogenate of larvae midgut tissue was added to new test tubes. The final volume was changed by adding 2.90 ml of 50 mM Tris and 10 mM EDTA, pH 8.2, and then mixing in 100 μl of pyrogallol solution. A UV–visible spectrophotometer was used to measure the absorbance at 440 nm.

#### Artificial soil assay

Artificial soil for use in the artificial soil toxicity assay was comprised of 27% kaolinite clay, 17% sphagnum peat, and 77% soil in accordance with OECD (1984). Three drops of CaCO_3_ were added to regulate the soil pH to 6.0 ± 0.2. Soil water content was maintained within the range of 30 ± 2%. Soil samples were prepared by adding different concentrations of fungal conidia as follows: 2.5 × 10^3^, 2.5 × 10^4^, 2.5 × 10^5^, 2.5 × 10^6,^ and 2.5 × 10^7^ conidia/kg on the basis of dry weight. Fifteen mature *E*. *eugeniae* earthworms were transferred to the 1 kg test substrate with different concentrations in test containers, which were covered tightly to prevent the escape of worms. A control group was set up involving the artificial substrate in the absence of a fungal conidia solution. Each replicate contained 15 adults of *E*. *eugeniae,* and was replicated in triplicate. The mortality of earthworms was calculated after 3, 6, 9, and 12 days of exposure time.

#### Histopathological study

*E. eugeniae* were exposed to various concentrations of fungal conidia (treatment group) or no fungal conidia (control group) for 12 days. After 12 days of treatment, control and fungal conidia treated earthworms were fixed in 3% formalin for 3 h at 4°C. Formalin blocks were chilled at 25°C for 3 h, then sliced into 2.5-mm-thick ribbons with 0.5-mm ribbons using a microtome (Leica, Germany). *E*. *eugeniae* was stained with Ehrlich’s hematoxylin and eosin. After staining, slides were viewed under a light microscope at 40× magnification (Olympus CH20i/Thailand).

### Statistical analysis

Mortality of *S. frugiperda and E. eugeniae* was calculated using the Abbott formula (Abbott, 1925). A one-way ANOVA was used to calculate the variances between treatments, and the Tukey’s HSD test was done to categorise the homogeneous types of data sets using SPSS software. In all experiments, the significance level was reported as *p* = 0.05.

## Results

### Morphological and molecular identification

**Figure 2:**
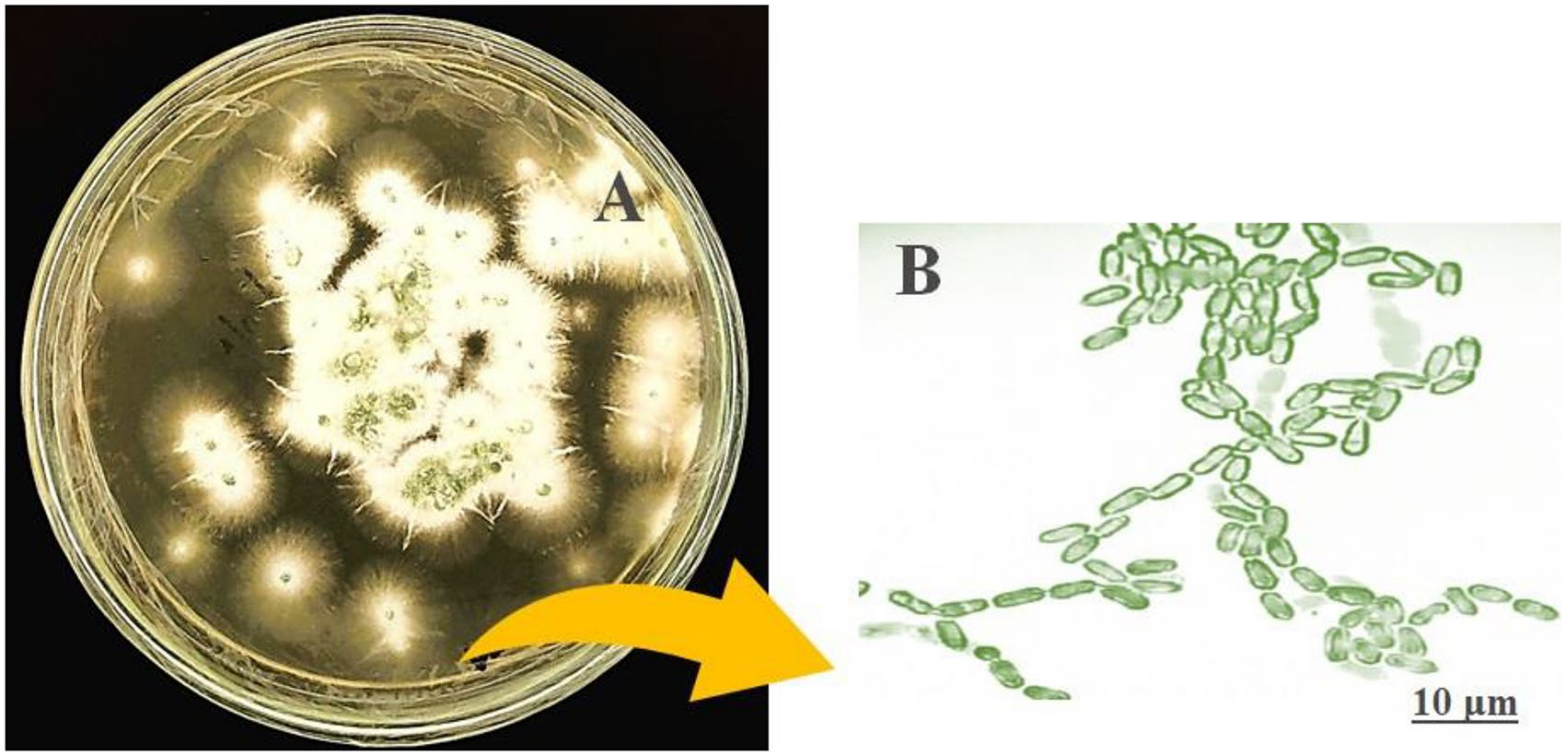
Seven day old culture of *Metarhizium majus* fungi culture on PDA media. **(A)** *Metarhizium majus* culture, **(B)** Fungal conidial morphological structures at 40× magnification under light microscope.

**Figure 3:**
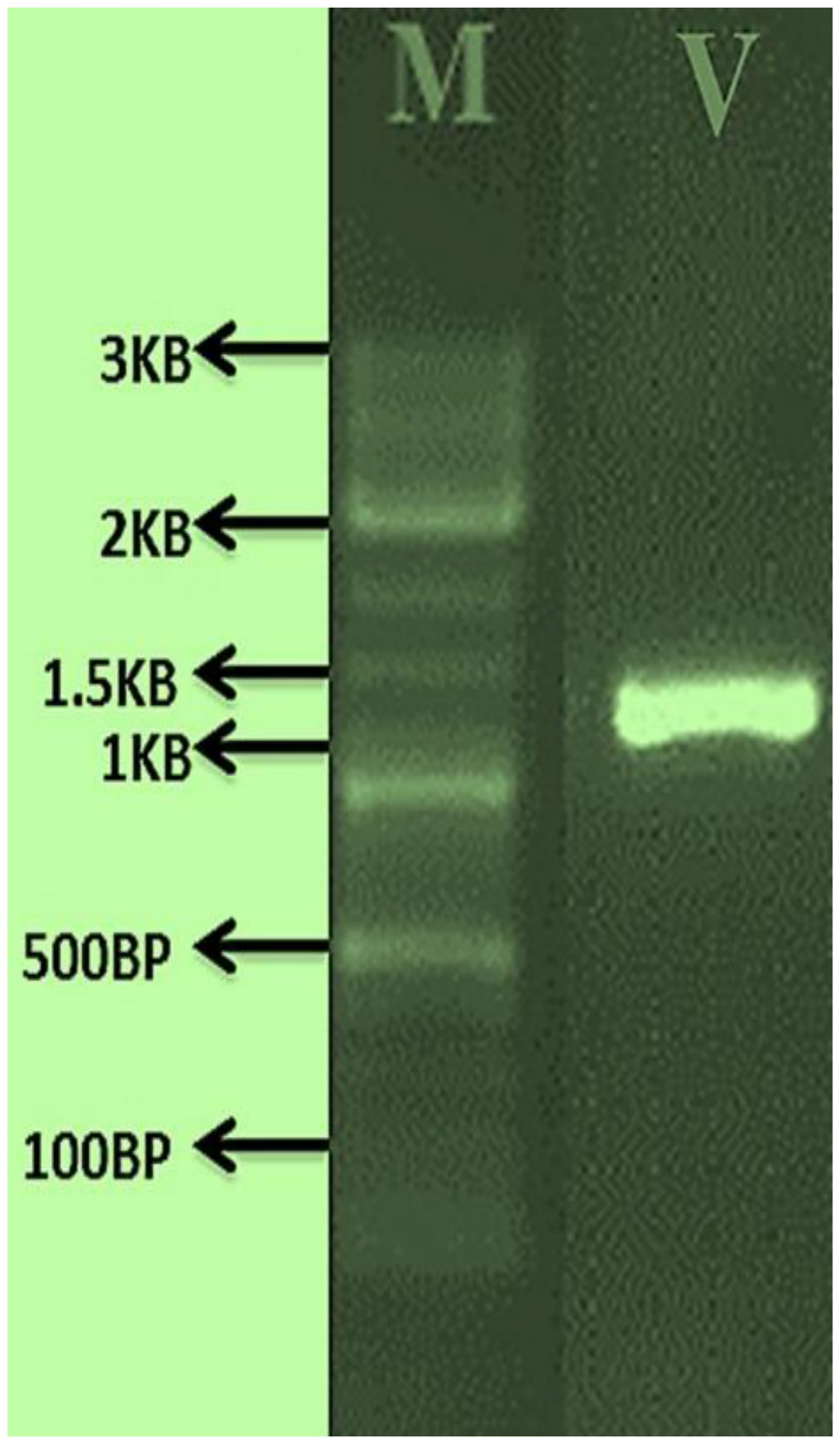
PCR amplified fungal genomic DNA fragments. V: *M. majus* DNA fragment size range is 1.3 KP, M: 3 KB Marker.

**Figure 4:**
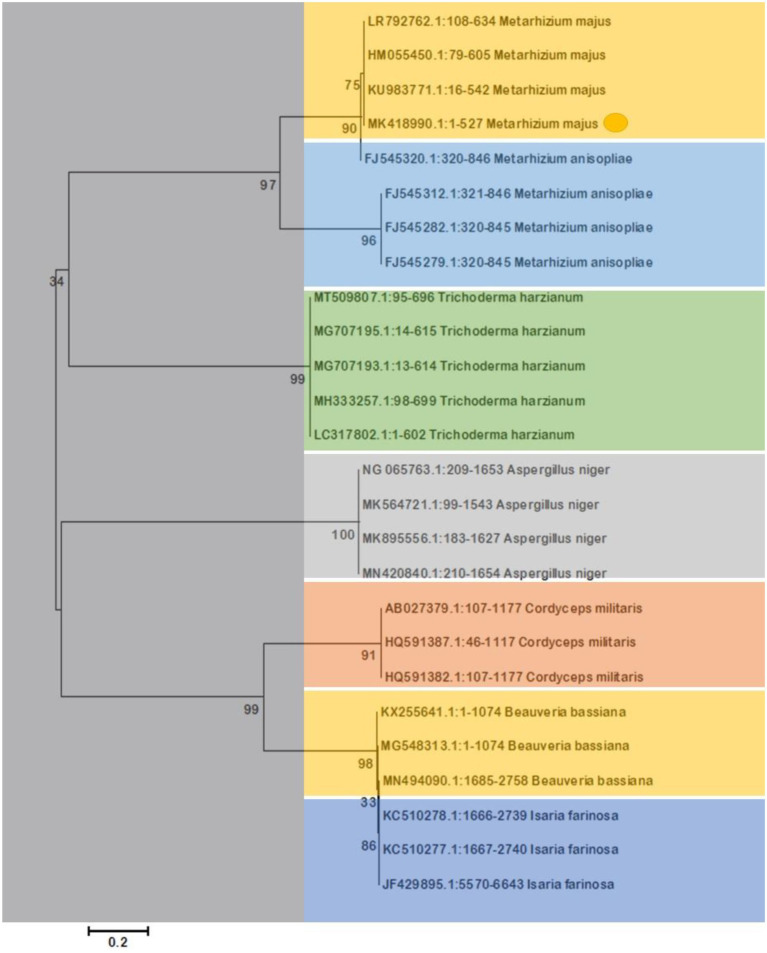
The Neighbor-Joining tree method was used to make a phylogenetic tree of the stages of evolution of entomopathogenic fungi that had been isolated. Our isolated fungal strains showed 100% similarity with *Metarhizium majus*.

The morphological features of isolated entomopathogenic fungi was shown to be light green in colour and rod-like in shape, and produced green conidia (Figure 2). Entomopathogenic fungal genomic DNA was amplified in an optimised state using a universal primer by the 18s rDNA gene, and the amplified DNA fragments were examined using a gel documentation unit. The DNA fragment range obtained was 1,200 bp (Figure 3). The sequence quality of the amplified DNA molecules was evaluated. The fungal DNA sequence was submitted to the GenBank database (NCBI). *M. majus* has the accession number (MK418990.1). The results of the 18 s rRNA sequence BLAST search revealed a perfect match with previously reported *Metarhizium majus* cultures (Nishi et al., 2015). The neighbour-joining tree method was used to determine the evolutionary closeness of isolated entomopathogenic fungi (Figure 4).

### Larval bioassay

**Figure 5:**
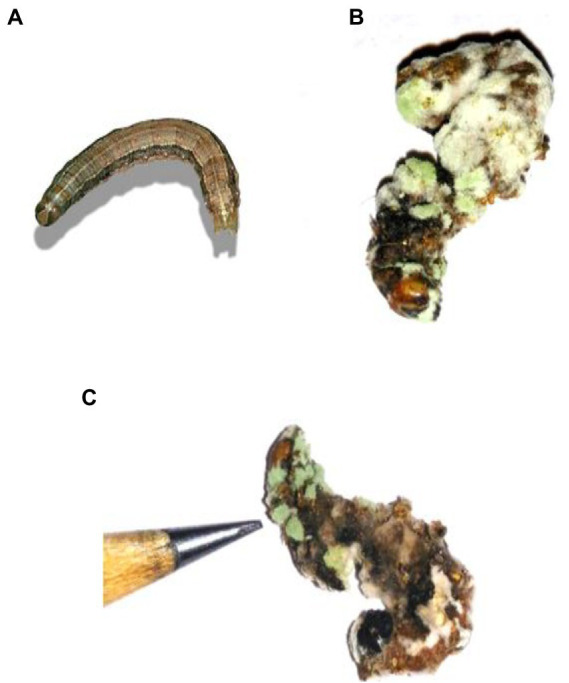
Cadaver of *S. frugiperda* after treatment with *M. majus* following 3, 6, 9, and 12 days of treatment under laboratory condition. **(A)** Control (without any conidia); **(B,C)** treatment with *M. majus* with results showing after 6 days of treatment. Fungi conidia had completely covered the cadaver of *S. frugiperda*.

**Figure 6:**
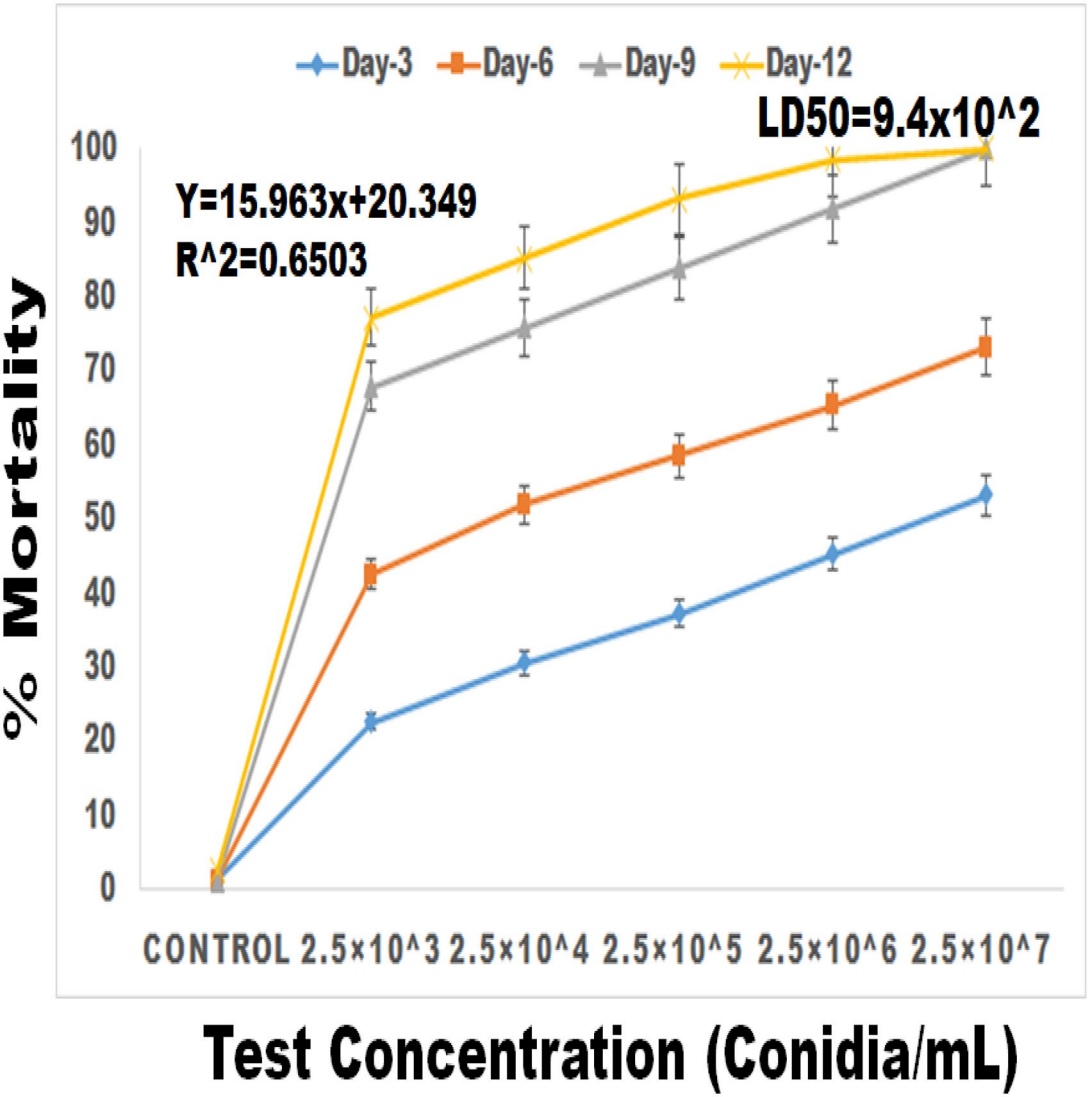
Percentage mortality of *S. frugiperda* after treatment of *M. majus* conidia for 3, 6, 9, and 12 days under laboratory condition. A *M. majus* conidia treatment at 3, 6, 9, and 12 days of treatment.

Entomopathogenic fungal conidia caused significant larvicidal activity against third instar larvae of *S. frugiperda*. *S. frugiperda* larvae displayed 53% (df:5; *F*_(5,12)_ = 34.576; *p* < 0.01), 73% (df:5; F_(5,12)_ = 58.147; *p* < 0.01), 100% (df:5; F_(5,12)_ = 87.265; *p* < 0.01) and 100% (df:5; F_(5,12)_ = 201.226; *p* < 0.01) mortality following 3, 6, 9, and 12 days of treatment, respectively (Figures 5, 6). After 12 days of fungal treatment, entomopathogenic fungi proliferated both inside and outside the insect cadaver (Figure 5). In a similar study, entomopathogenic fungi *Metarhizium majus* fungal conidia demonstrated high insecticidal activity against the coleopteran pests *Holotricha serrata* and *Oryctes rhinoceros* (Velavan et al., 2017).

### *Spodoptera frugiperda* larval enzyme response to fungal infection

**Figure 7:**
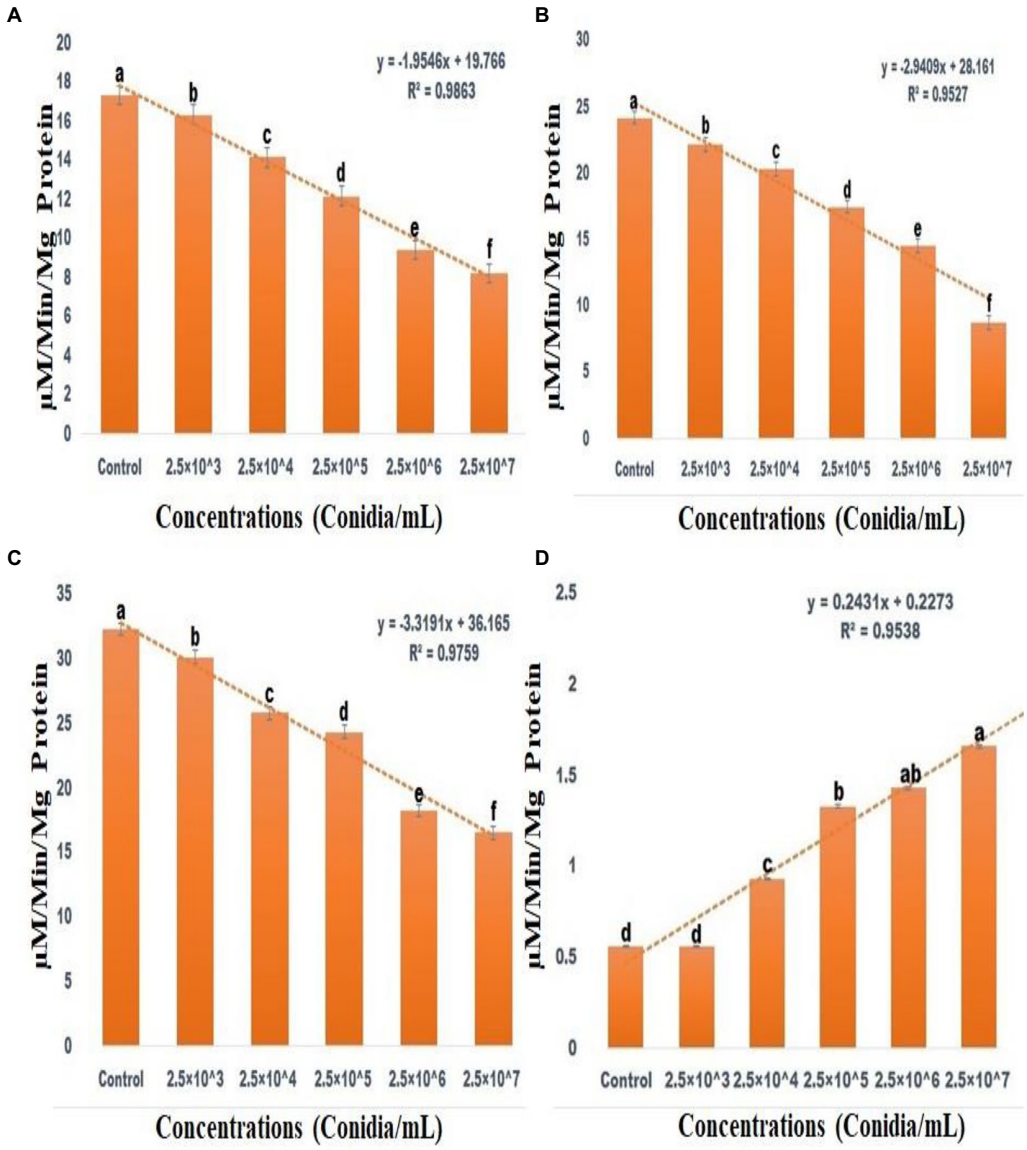
Acid Phosphatase activity **(A)**, Alkaline Phosphatase activity **(B)**, CAT assay **(C)**, Superoxide dismutase activities **(D)**. According to the Tukey test at *p =* 0.05, statistical values followed by the same letter do not differ significantly (one-way ANOVA).

ACP (F_(5,12)_ = 2154.806; df-5; *p* < 0.01), ALP (F_(5,12)_ = 2229.862; df-5; *p <* 0.01) and CAT (F_(5,12)_ = 4741.160; df-5; *p <* 0.01) activity were all significantly reduced after 3 days of entomopathogenic fungal conidia treatment when compared to the control group (Figures 7A–C). In contrast, SOD activity significantly increased after 3 days of entomopathogenic fungal conidia treatment when compared with the control group (F_(5,12)_ = 47.840; df-5; *p <* 0.01) (Figure 7D). A similar level of enzyme activity was observed when entomopathogenic fungi *Metarhizium flavoviride* was applied to *Spodoptera litura larvae* (Vivekanandhan et al., 2022d).

### *Eudrilus eugeniae* toxicity assay

Treatment of the terrestrial soil bioindicator species *E. eugeneae* with various fungal conidial concentrations revealed no lethal toxicity effects. No mortality was observed after 12 days of treatment. According to Zimmermann (2007), the entomopathogenic fungi *Beauveria bassiana* and *Beauveria brongniartii* have no sublethal toxicity effect on non-target earthworm species.

### Histopathology of *Eudrilus eugeniae*

**Figure 8:**
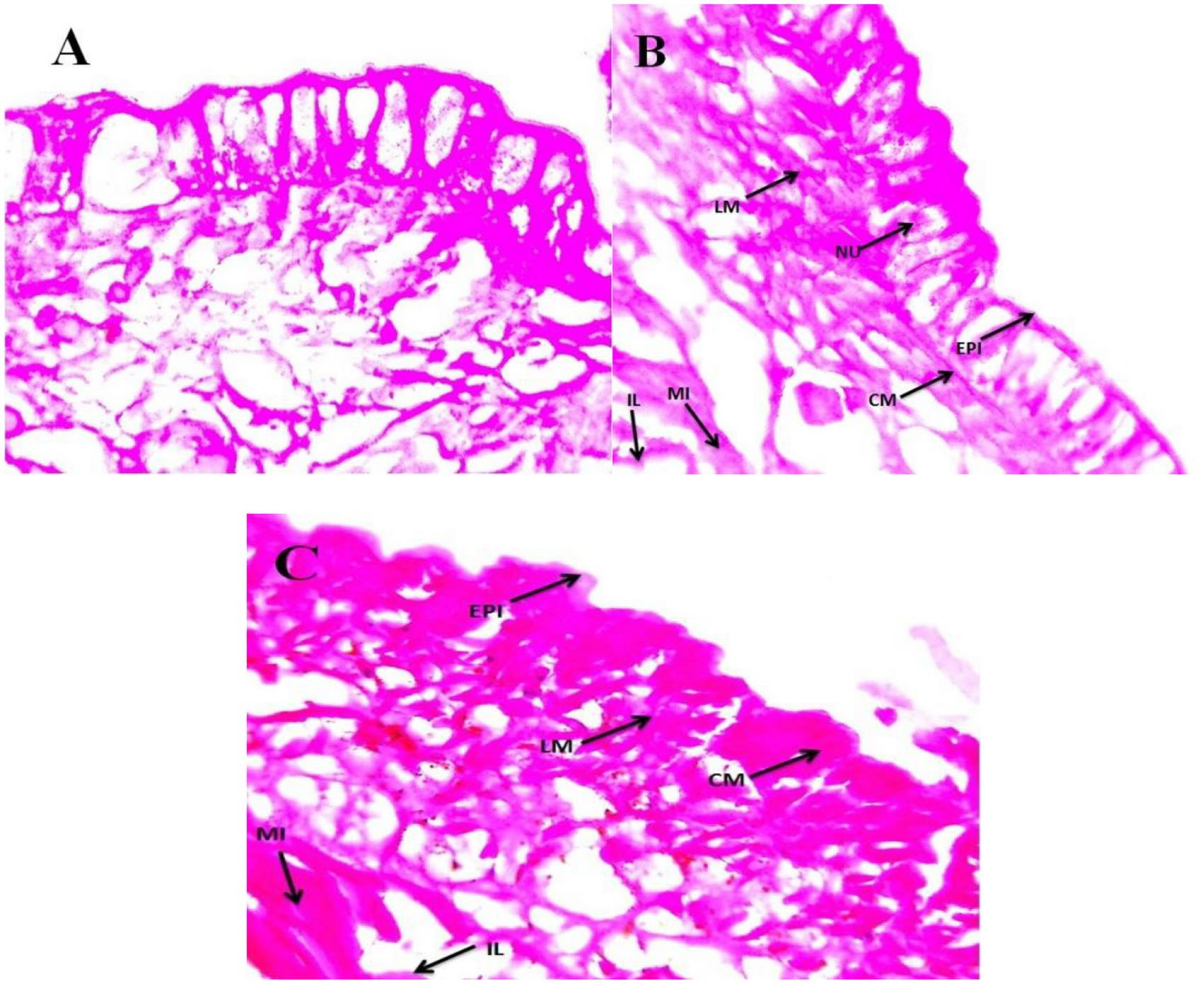
*Eudrilus eugeniae* cross sections magnified at 40× under a light microscope. **(A)** Control earthworm tissue (no treatment with *M. majus*); **(B,C)** earthworm tissues treated with *M. majus*. No histopathological changes were observed in the intestinal lumen and intestine of treated earthworms when compared with control earthworms and no fungal conidia were observed in the mid gut cross section. EPI, epidermis; CM, circular muscle; LM, longitudinal muscle; SE, setae; CO, coelom; MI, mitochondrion; IL, intestinal lumen.

Sub-lethal toxicity was evaluated in *E*. *eugeniae* tissues after 12 days of entomopathogenic fungal conidia treatment. Results showed that fungal conidia induced no damage to gut tissues, namely, the lumen and epithelial cells, nucleus, setae, coelom, mitochondria, and muscles (Figure 8). The tissues of earthworms treated with the entomopathogenic fungus *M. majus* conidia did not differ from the tissues of control earthworm. Similarly, to this study, entomopathogenic fungi *Beauveria bassiana* had no histopathological effect on the earthworm *Eisenia fetida* (Zhou et al., 2021).

## Discussion

Entomopathogenic fungi play an important role in controlling insect pests in an integrated pest management program. The previous study provided a thorough investigation into the use of *M. majus*, first isolated in 2021 (Kwak et al., 2021), in the control of *S. frugiperda*. Results revealed that bag-formulated *M. majus* conidia cause significant mortality in *S. frugiperda* larvae after 12 days of treatment, whilst leaving the non-target bioindicator species *E. fetida* unharmed.

Entomopathogenic fungi have been shown to induce mortality across all developmental stages of major insect pests under both laboratory and field conditions and thus represent an important tool in the control of insect pests (Vivekanandhan et al., 2021). *M. anisopliae*, *B. bassiana*, *V. lecanii*, and *F. oxysporum* fungal conidia, for example, have been shown to be highly virulent against several medical and agricultural insect pests including *Anopheles stephensi*, *Aedes aegypti*, *Culex quinquefasciatus*, *Tenebrio molitor* and *Spodoptera litura* (e.g., Roberts and Humber, 1981; Rai et al., 2014; Sujeetha and Sahayaraj, 2014; Islam et al., 2021). In addition, spores of *M. anisopliae*, *B. bassiana*, *V. lecanii*, *C. tropicum*, *C. lobatum*, and *L. giganteum* induce significant mortality in larvae of the mosquito *A. aegypti*, *A. stephensi*, and *C. quinquefasciatus* (Darbro et al., 2011; Singh and Prakash, 2014; Bharathi et al., 2022). In the present study, *M. majus* exhibited distinct morphological and molecular characteristics in line with previous studies (Nishi et al., 2015; Sivakumar et al., 2020; Kwak et al., 2021; Mathulwe et al., 2021). *M. majus* is known to display cylindrical to ellipsoid characteristics, forming candelabrum-like arrangements that create compact conidiophores in a hymenial layer. Mature conidia colonies, which are dark green in color, form chains of equal length within clusters (Mathulwe et al., 2021). Furthermore, bag-formulated *M. majus* conidia induced significant larvicidal activity against third instar larvae of *S. frugiperda*, with 100% mortality achieved within 9 days. Dose-dependent larvicidal activity was observed, with the highest larvicidal *activity* achieved at a dose of 2.5 × 10^7^ conidia/ml. *M. majus* fungal pathogenicity and conidial growth were visible on the dead cadavers of *S. frugiperda*, while in control samples, insect larvae were in good physical shape and no fungal infection was observed. These findings support previous research on species of the genus *Spodoptera*, whereby *M. rileyi* caused significant insecticidal activity on *S. litura* in 10 days (Dutta et al., 2014). Likewise, Krishnaveni et al. (2016) reports 80% mortality with a grain formulation of *M. rileyi* against third instar larvae of *S. litura* in laboratory experiments.

Previous research has found that, among the tested lepidopteran insects, *B. bassiana* and *M. anisopliae* produce the greatest insecticidal effect against *S. frugiperda* (Wraight et al., 2010). The fungi conidia of *B. bassiana* resulted in 96.6% larval mortality when isolated from cadavers of mosquitoes (García-Munguía et al., 2011). *B. bassiana* strains, isolated from dead larvae of *S. frugiperda* caused 74% larval mortality (Cruz-Avalos et al., 2019). Likewise, this present study showed that entomopathogenic fungi *M. majus* conidia caused high larvicidal activity against *S. frugiperda* within 9 days under laboratory conditions. Similarly, *M. anisopliae* and *B. bassiana* spores display insecticidal activity against disease-transmitting mosquito *Aedes* and *Culex* species (Ravindran et al., 2015; Lee et al., 2019; Hamama et al., 2022).

Enzymatic analysis revealed altered ACP, ALP, catalase, and SOD enzyme activity in third instar larvae of *S. frugiperda* infected with *M. majus*, thus indicating that the entomopathogenic fungi alters the insect immune system to enable infection. Enzyme levels changed with dose-dependent activity in response to entomopathogenic fungi conidia treatment compared to a control Here, larval ACP, ALP, and catalase enzyme levels significantly decreased following fungi conidia treatment. In contrast, SOD enzyme levels significantly increased following fungi conidia treatment. Catalase (CAT) enzymes are critical in the defence against H_2_O_2_ entomopathogens and other toxicants. The findings indicate that the pathogenicity of *M. majus* fungi conidia reduce the level of CAT enzyme in *S. frugiperda* larvae. CAT enzymes are also important in the fight against oxidative stress. Wang and Granados (2000) discovered that when *S. litura* larvae are exposed to UV light, their CAT enzyme levels decrease. The excess ROS produced by the crude extract suppresses antioxidant enzymes. Extremely high levels of H_2_O_2_ have been shown to inhibit SOD enzymes by generating more hydroxyl radicals (Vivekanandhan et al., 2022b).

In the current study, *M. majus* conidia had no toxic effect and caused no mortality in the terrestrial soil bioindicator *E. eugeniae* following 12 days of treatment. This supports previous research which has reported that entomopathogenic fungi conidia and their secondary metabolites do not kill earthworms (Vivekanandhan et al., 2021, 2022a,b). The histopathological evaluation of *E. eugeniae* after 12 days of treatment with *M. majus* fungal conidia (2.5 × 10^7^ conidia/kg) showed that fungal conidia do not damage gut tissues, including lumen and epithelial cells, the nucleus, setae, coelom, mitochondria, and muscles, and that entomopathogenic fungi are unable to penetrate the earthworm epidermis. It is thought that the epidermal mucus of the earthworm *E. fetida* is capable of destroying the entomopathogenic fungi *B. bassiana* conidia, thus preventing infection (Zhou et al., 2021). It is possible that the epidermal mucus is also capable of killing *M. majus*, thus preventing infection of the earthworm as observed in the current study.

## Conclusion

In conclusion, *M. majus* conidia caused high insecticidal efficacy against third instar larvae of *S. frugiperda* in 2.5 × 10^7^ conidia/ml concentration, producing 100% mortality within 9 days. No sublethal toxicity was observed in *E. eugeniae* after 12 days of treatment. Histopathological evaluation of *E. eugeniae* exposed to *M. majus* conidia showed no toxicity effects at 12 days post-exposure. *M. majus* exposure causes a physiological response in *S. frugiperda* that results in lower levels of ALP and ACP, CAT enzymes in larvae. Furthermore, after 3 days of treatment, the level of the SOD enzyme increases. The findings suggest that *M. majus* fungal infection of *S. frugiperda* larvae affects insect immune function directly, with larval immune function being significantly reduced. Larvicidal activity indicates that *M. majus* fungi spores can kill *S. frugiperda* larvae in 3 days. Overall, our findings indicate that *M. majus* conidia has potential in the control of the agricultural pest *S. frugiperda* while being less toxic to the non-target organism *E. eugeniae*.

## Data availability statement

The datasets presented in this study can be found in online repositories. The names of the repository/repositories and accession number(s) can be found in the article/supplementary material.

## Author contributions

VP, PK, and SK: conceptualization. VP and SK: data observation, methodology, and writing – original draft. VP: formal analysis and supervision. VP and PK: investigation. SK: resources and visualization. LA, DE, RM, VP, and SK: validation. VP, LA, DE, RM, SP, RG, PK, and SK: writing – review and editing. All authors contributed to the article and approved the submitted version.

## Funding

This research work was partially supported by Chiang Mai University.

## Conflict of interest

The authors declare that the research was conducted in the absence of any commercial or financial relationships that could be construed as a potential conflict of interest.

## Publisher’s note

All claims expressed in this article are solely those of the authors and do not necessarily represent those of their affiliated organizations, or those of the publisher, the editors and the reviewers. Any product that may be evaluated in this article, or claim that may be made by its manufacturer, is not guaranteed or endorsed by the publisher.

## Footnotes

^1^ www.dropdata.net

## References

[ref1] Abbott W. S. (1925). A method of computing the effectiveness of an insecticide. J. Econ. Entomol. 18, 265–267. doi: 10.1093/jee/18.2.265a, PMID: 36749608

[ref4] Asakura K. (1978). Phosphatase activity in the larva of the euryhaline mosquito, Aedes togoi Theobald, with special reference to sea-water adaptation. J. Exp. Mar. Biol. Ecol. 31, 325–337. doi: 10.1016/0022-0981(78)90067-9

[ref5] Aw K. M. S. Hue S. M. (2017). Mode of infection of *Metarhizium* spp. fungus and their potential as biological control agents. J. Fungi 3:30. doi: 10.3390/jof3020030, PMID: 29371548 PMC5715920

[ref6] Bartlett M. C. Jaronski S. T. (1988). Mass production of entomogenous fungi for biological control of insects. Fungi Biological Control Syst 1988, 61–85.

[ref7] Batta Y. A. (2003). Production and testing of novel formulations of the entomopathogenic fungus *Metarhizium anisopliae* (Metschinkoff) Sorokin (Deuteromycotina: Hyphomycetes). Crop Prot. 22, 415–422. doi: 10.1016/S0261-2194(02)00200-4

[ref8] Bharathi N. S. Mahendran P. Sujatha K. Ashokraj S. Rabeesh T. P. (2022). Pathogenic potential of *Metarhizium anisopliae* and *Lecanicillium longisporum* on tea mosquito bug, *Helopeltis theivora* Waterhouse (Hemiptera: Miridae). J. Basic Appl. Zool. 83, 1–9. doi: 10.1186/s41936-022-00297-4

[ref10] Canassa F. Esteca F. C. Moral R. A. Meyling N. V. Klingen I. Delalibera I. (2020). Root inoculation of strawberry with the entomopathogenic fungi *Metarhizium robertsii* and *Beauveria bassiana* reduces incidence of the two spotted spider mite and selected insect pests and plant diseases in the field. J. Pest. Sci. 93, 261–274. doi: 10.1007/s10340-019-01147-z

[ref12] Cruz-Avalos A. M. Bivián-Hernández M. D. L. Á. Ibarra J. E. Del Rincón-Castro M. C. (2019). High virulence of Mexican entomopathogenic fungi against fall armyworm, (Lepidoptera: Noctuidae). J. Econ. Entomol. 112, 99–107. doi: 10.1093/jee/toy343, PMID: 30383250

[ref13] Darbro J. M. Graham R. I. Kay B. H. Ryan P. A. Thomas M. B. (2011). Evaluation of entomopathogenic fungi as potential biological control agents of the dengue mosquito, *Aedes aegypti* (Diptera: Culicidae). Biocontrol Sci. Tech. 21, 1027–1047. doi: 10.1080/09583157.2011.597913, PMID: 33122748

[ref15] De Faria M. R. Wraight S. P. (2007). Mycoinsecticides and mycoacaricides: a comprehensive list with worldwide coverage and international classification of formulation types. Biol. Control 43, 237–256. doi: 10.1016/j.biocontrol.2007.08.001

[ref16] Dutta P. Patgiri P. Pegu J. Kaushik H. Boruah S. (2014). First record of *Nomuraea rileyi* (Farlow) Samson on *Spodoptera litura* Fabricius (Lepidoptera: Noctuidae) from Assam, India. Curr Biotica 8, 187–190.

[ref17] Feng M. G. Poprawski T. J. Khachatourians G. G. (1994). Production, formulation and application of the entomopathogenic fungus *Beauveria bassiana* for insect control: current status. Biocon Sci Technol 4, 3–34. doi: 10.1080/09583159409355309

[ref21] García-Munguía A. M. Garza-Hernández J. A. Rebollar-Tellez E. A. Rodríguez-Pérez M. A. Reyes-Villanueva F. (2011). Transmission of *Beauveria bassiana* from male to female *Aedes aegypti* mosquitoes. Parasit. Vectors 4, 1–6. doi: 10.1186/1756-3305-4-24 21352560 PMC3051917

[ref22] Hall S., (1981). Physiology of conidial fungi. Biology of conidial fungi, 2, 417–457.

[ref23] Hall T. A. (1999). BioEdit: a user-friendly biological sequence alignment editor and analysis program for Windows 95/98/NT. Nucleic Acids Symp. Ser. 41, 95–98.

[ref24] Hamama H. M. Zyaan O. H. Ali O. A. A. Saleh D. I. Elakkad H. A. El-Saadony M. T.. (2022). Virulence of entomopathogenic fungi against *Culex pipiens*: Impact on biomolecules availability and life table parameters. Saudi J. Biol. Sci. 29, 385–393. doi: 10.1016/j.sjbs.2021.08.103, PMID: 35002434 PMC8716910

[ref26] Islam W. Adnan M. Shabbir A. Naveed H. Abubakar Y. S. Qasim M.. (2021). Insect-fungal-interactions: A detailed review on entomopathogenic fungi pathogenicity to combat insect pests. Microb. Pathog. 159:105122. doi: 10.1016/j.micpath.2021.105122, PMID: 34352375

[ref27] Kim J. J. Jeong G. Han J. H. Lee S. (2013). Biological control of aphid using fungal culture and culture filtrates of *Beauveria bassiana*. Mycobiology 41, 221–224. doi: 10.5941/MYCO.2013.41.4.221, PMID: 24493943 PMC3905126

[ref28] Krishnaveni S. Manjula K. Murali Krishna T. Ahammad S. K. (2016). Preparation of grain formulations of *Nomuraea rileyi* (Farlow) Samson and testing virulence against 3rd instar of *Spodoptera litura*. Bioscan 11, 185–187.

[ref29] Kumar K. K. Sridhar J. Murali-Baskaran R. K. Senthil-Nathan S. Kaushal P. Dara S. K.. (2019). Microbial biopesticides for insect pest management in India: Current status and future prospects. J. Invertebr. Pathol. 165, 74–81. doi: 10.1016/j.jip.2018.10.008, PMID: 30347206

[ref30] Kwak K. W. Aktaruzzaman M. Kim E. Kim S. Y. Hong S. B. Park J. Y.. (2021). Identification and characterization of *Metarhizium majus* isolated from the edible insect *Protaetia brevitarsis* in Korea. Entomol. Res. 51, 602–609. doi: 10.1111/1748-5967.12554

[ref31] Lee J. Y. Woo R. M. Choi C. J. Shin T. Y. Gwak W. S. Woo S. D. (2019). Beauveria bassiana for the simultaneous control of *Aedes albopictus* and *Culex pipiens* mosquito adults shows high conidia persistence and productivity. AMB Express 9, 1–9. doi: 10.1186/s13568-019-0933-z 31865499 PMC6925604

[ref33] Liu Y. C. Ni N. T. Chang J. C. Li Y. H. Lee M. R. Kim J. S.. (2021). Isolation and selection of entomopathogenic fungi from soil samples and evaluation of fungal virulence against insect pests. J. Vis. Exp. 175:e62882. doi: 10.3791/62882-v 34661569

[ref35] Marklund S. Marklund G. (1974). Involvement of the superoxide anion radical in the autoxidation of pyrogallol and a convenient assay for superoxide dismutase. Eur. J. Biochem. 47, 469–474. doi: 10.1111/j.1432-1033.1974.tb03714.x, PMID: 4215654

[ref36] Mathulwe L. L. Malan A. P. Stokwe N. F. (2021). A review of the biology and control of the obscure mealybug, *Pseudococcus viburni* (Hemiptera: Pseudococcidae), with special reference to biological control using entomopathogenic fungi and nematodes. Afr. Entomol. 29, 1–16. doi: 10.4001/003.029.0001

[ref39] Nishi O. Iiyama K. Yasunaga-Aoki C. Shimizu S. (2015). Phylogenetic status and pathogenicity of *Metarhizium majus* isolated from a fruit beetle larva in Japan. Mycol. Prog. 14, 1–10.

[ref130] OECD. (1984). Guideline for testing of chemicals. Earthworm acute toxicity. In OECD, ed. OECD, Paris.

[ref40] Pham T. A. Kim J. J. Mm S. G. Kim K. (2009). Production of blastospore of entomopathogenic *Beauveria bassiana* in a submerged batch culture. Mycobiology 37, 218–224. doi: 10.4489/MYCO.2009.37.3.218, PMID: 23983537 PMC3749392

[ref41] Pittarate S. Rajula J. Rahman A. Vivekanandhan P. Thungrabeab M. Mekchay S.. (2021). Insecticidal effect of zinc oxide nanoparticles against *Spodoptera frugiperda* under laboratory conditions. Insects 12:1017. doi: 10.3390/insects12111017, PMID: 34821816 PMC8618014

[ref42] Rai D. Updhyay V. Mehra P. Rana M. Pandey A. K. (2014). Potential of entomopathogenic fungi as biopesticides. Indian J. Sci. Res. Technol. 2, 7–13.

[ref43] Rambaut A. (2009). FigTree. Tree Figure Drawing Tool Version 1.3.1. Edinburgh: University of Edinburgh.

[ref44] Rao C. N. Shivankar V. J. Singh S. (2006). Citrus mealybug (*Planococcuscitri risso*) management – a review. Agri Rev 27, 142–146.

[ref45] Ravindran K. Rajkuberan C. Prabukumar S. Sivaramakrishnan S. (2015). Evaluation of pathogenicity of *Metarhizium anisopliae* TK-6 against developmental stages of *Aedes aegypti* and *Culex quinquefasciatus*. J Pharm Biol Eval 2, 188–196.

[ref47] Roberts D. W. Humber R. A. (1981). Entomogenous fungi. Biol. Conidial Fungi 2:e236

[ref48] Rwomushana I. (2019). *Spodoptera frugiperda* (fall armyworm). Invasive Species Compendium:29810.

[ref49] Saitou N. Nei M. (1987). The neighbor-joining method: a new method for reconstructing phylogenetic trees. Mol. Biol. Evol. 4, 406–425. doi: 10.1093/oxfordjournals.molbev.a040454, PMID: 3447015

[ref50] Schrank A. Vainstein M. H. (2010). *Metarhizium anisopliae* enzymes and toxins. Toxicon 56, 1267–1274. doi: 10.1016/j.toxicon.2010.03.008, PMID: 20298710

[ref51] Shah P. A. Pell J. K. (2003). Entomopathogenic fungi as biological control agents. Appl. Microbiol. Biotechnol. 61, 413–423. 12764556 10.1007/s00253-003-1240-8

[ref52] Shylesha A. N. Jalali S. K. Gupta A. N. K. I. T. A. Varshney R. I. C. H. A. Venkatesan T. Shetty P. R. A. D. E. E. K. S. H. A.. (2018). Studies on new invasive pest *Spodoptera frugiperda* (JE Smith) (Lepidoptera: Noctuidae) and its natural enemies. J Bio Con 32, 1–7.

[ref53] Singh G. Prakash S. (2014). New prospective on fungal pathogens for mosquitoes and vectors control technology. J. Mosquito Res. 4, 36–52.

[ref54] Singh D. Raina T. K. Singh J. (2017). Entomopathogenic fungi: An effective biocontrol agent for management of insect populations naturally. J. Pharm. Sci. Res. 9:833.

[ref55] Sivakumar T. Jiji T. Naseema A. (2020). Effect of pesticides used in banana agro-system on entomopathogenic fungus, *Metarhizium majus* Bisch, Rehner and Humber. Int. J. Trop. Insect Sci. 40, 283–291. doi: 10.1007/s42690-019-00080-z

[ref57] Sujeetha J. A. R. P. Sahayaraj K. (2014). “Role of entomopathogenic fungus in pest management” in Basic and Applied Aspects of Biopesticides (New Delhi: Springer), 31–46.

[ref59] Tamura K. Nei M. Kumar S. (2004). Prospects for inferring very large phylogenies by using the neighbor-joining method. Proc. Natl. Acad. Sci. U. S. A. 101, 11030–11035. doi: 10.1073/pnas.0404206101, PMID: 15258291 PMC491989

[ref60] Tamura K. Peterson D. Peterson N. Stecher G. Nei M. Kumar S. (2011). MEGA5: molecular evolutionary genetics analysis using maximum likelihood, evolutionary distance, and maximum parsimony methods. Mol. Biol. Evol. 10, 2731–2739. doi: 10.1093/molbev/msr121 PMC3203626 21546353

[ref61] Thanakitpipattana D. Tasanathai K. Mongkolsamrit S. Khonsanit A. Lamlertthon S. Luangsa-Ard J. J. (2020). Fungal pathogens occurring on Orthopterida in Thailand. Persoonia Mol. Phylogeny Evol. Fungi 44, 140–160. doi: 10.3767/persoonia.2020.44.06, PMID: 33116339 PMC7567961

[ref63] Velavan V. Rangeshwaran G. S. R. Sundararaj R. Sasidharan T. (2017). *Metarhizium majus* and *Metarhizium robertsii* show enhanced activity against the coleopteran pests *Holotricha serrata* and *Oryctes rhinoceros*. J. Biol. Control. 31, 135–145.

[ref64] Vivekanandhan P. Bedini S. Shivakumar M. S. (2020). Isolation and identification of entomopathogenic fungus from Eastern Ghats of South Indian forest soil and their efficacy as biopesticide for mosquito control. Parasitol. Int. 76:102099. doi: 10.1016/j.parint.2020.102099, PMID: 32169659

[ref65] Vivekanandhan P. Swathy K. Alford L. Pittarate S. Subala S. P. R. R. Mekchay S.. (2022d). Toxicity of *Metarhizium flavoviride* conidia virulence against *Spodoptera litura* (lepidoptera: noctuidae) and its impact on physiological and biochemical activities. Sci. Rep. 12:16775. 36202839 10.1038/s41598-022-20426-x PMC9537412

[ref66] Vivekanandhan P. Swathy K. Murugan A. C. Krutmuang P. (2022c). Insecticidal efficacy of *Metarhizium anisopliae* derived chemical constituents against disease-vector mosquitoes. J Fungi 8:300. 10.3390/jof8030300 PMC8950813 35330302

[ref67] Vivekanandhan P. Swathy K. Shivakumar M. S. (2022a). Identification of insecticidal molecule aucubin from *Metarhizium anisopliae* ethyl acetate crude extract against disease mosquito vector. Int. J. Trop. Insect Sci. 18, 3303–3318.

[ref68] Vivekanandhan P. Swathy K. Shivakumar M. S. (2022b). Stability of insecticidal molecule aucubin and their toxicity on *Anopheles stephensi*, *Aedes aegypti*, *Culex quinquefasciatus* and *Artemia salina*. Int. J. Trop. Insect Sci. 42, 3403–3417.

[ref69] Vivekanandhan P. Swathy K. Thomas A. Kweka E. J. Rahman A. Pittarate S.. (2021). Insecticidal efficacy of microbial-mediated synthesized copper nano-pesticide against insect pests and non-target organisms. Int. J. Environ. Res. Public Health 18:10536. doi: 10.3390/ijerph181910536, PMID: 34639837 PMC8508597

[ref140] Wang P. Granados R. R. (2000). Calcofluor disrupts the midgut defense system in insects. Insect Biochemistry and Molecular Biology 30, 135–143. 10696589 10.1016/s0965-1748(99)00108-3

[ref120] Wang Y. Oberley L. W. Murhammer D. W. (2001). Evidence of oxidative stress following the viral infection of two lepidopteran insect cell lines. Free Radical Biology and Medicine 31, 1448–1455. 11728817 10.1016/s0891-5849(01)00728-6

[ref72] Wasuwan R. Phosrithong N. Promdonkoy B. Sangsrakru D. Sonthirod C. Tangphatsornruang S.. (2021). The fungus *Metarhizium* sp. BCC 4849 is an effective and safe mycoinsecticide for the management of spider mites and other insect pests. Insects 13:42. 35055885 10.3390/insects13010042 PMC8780889

[ref73] Wraight S. P. Jackson M. A. De Kock S. L. (2001). 10 Production, Stabilization and Formulation of Fungal Biocontrol Agents, United States: Fungi as Biocontrol Agents, 253.

[ref74] Wraight S. P. Ramos M. E. Avery P. B. Jaronski S. T. Vandenberg J. D. (2010). Comparative virulence of *Beauveria bassiana* isolates against lepidopteran pests of vegetable crops. J. Invertebr. Pathol. 103, 186–199. doi: 10.1016/j.jip.2010.01.001, PMID: 20060396

[ref75] Wu J. Li J. Zhang C. Yu X. Cuthbertson A. G. Ali S. (2020). Biological impact and enzyme activities of *Spodoptera litura* (Lepidoptera: Noctuidae) in response to synergistic action of Matrine and *Beauveria brongniartii*. Front. Physiol. 11:584405. doi: 10.3389/fphys.2020.584405, PMID: 33224038 PMC7667252

[ref78] Xiao H. Ye X. Xu H. Mei Y. Yang Y. Chen X.. (2020). The genetic adaptations of fall armyworm *Spodoptera frugiperda* facilitated its rapid global dispersal and invasion. Mol Ecol Res 20, 1050–1068. doi: 10.1111/1755-0998.13182, PMID: 32359007

[ref83] Zhou X. Liang W. Zhang Y. Ren Z. Xie Y. (2021). Effect of earthworm *Eisenia fetida* epidermal mucus on the vitality and pathogenicity of *Beauveria bassiana*. Sci. Rep. 11, 1–11. 34230511 10.1038/s41598-021-92694-y PMC8260715

[ref85] Zimmermann G. (2007). Review on safety of the entomopathogenic fungi *Beauveria bassiana* and *Beauveria brongniartii*. Biocontrol Sci. Tech. 17, 553–596. doi: 10.1080/09583150701309006

